# Features of Female Offenders Remanded to a Tertiary Psychiatric Hospital for Assessment in Singapore

**DOI:** 10.1192/bjo.2026.11521

**Published:** 2026-06-30

**Authors:** Derrick Yeo

**Affiliations:** Institute of Mental Health, Singapore, Singapore. Lee Kong Chian School of Medicine, NTU, Singapore, Singapore

## Abstract

**Aims:**

Research and literature on female offenders are relatively scarce compared to their male counterparts. In Singapore, female offenders are remanded either at the only local psychiatric hospital (Institute of Mental Health) or at Changi Prison. The judge remands them for between two to three weeks at the first instance to determine if these offenders with suspected mental disorders have the ability to plead in court and to determine if their suspected mental health conditions have any contribution to their offending behaviour. Legal determinations of unsoundness of mind at the time of offence and fitness to plead at the time of trial carry significant implications for criminal responsibility and case disposition. The psychiatrist also has to instruct the court about the nature of the mental disorder, the risk of reoffending and recidivism and whether treatment is possible and also feasible in each remandee. This study reviews the psychiatric and legal characteristics of female remandees assessed at the Institute of Mental Health (IMH), Singapore, over a one-year period.

**Methods:**

A retrospective review of the electronic records was conducted by psychiatric doctors posted to the IMH. The details and medical records of the female remandees referred to IMH for forensic psychiatric assessment over one year was assessed by computer and a template for data extraction filled in manually. Demographic data, psychiatric diagnoses, offence characteristics, substance use history, and legal opinions on unsoundness of mind and fitness to plead were record, examined and analysed. Diagnostic distributions were analysed within the groups assessed as of unsound mind and/or without fitness to plead, with attention to overlap and discordance between these legal constructs.

**Results:**

Out of 280 female remandees assessed over 12 months, 12 (4%) were assessed as of unsound mind and 13 (≈5%) as unfit to plead. Schizophrenia was the most common diagnosis in both groups, followed by major neurocognitive disorder. Most individuals in both categories were charged with non-violent offences, and none had a documented substance use history. While there was substantial overlap between unsoundness of mind and unfitness to plead, several discordant cases were identified, illustrating the temporal and conceptual distinction between these determinations. Major neurocognitive disorder was particularly prominent among those unfit to plead, reflecting the central role of current cognitive capacity in trial competence. These findings challenge assumptions that forensic psychiatric morbidity is primarily associated with violence or substance misuse and highlight the relevance of endogenous psychiatric and neurocognitive conditions in female remandees.

**Conclusion:**

Female remandees assessed as of unsound mind or unfit to plead constitute a small but highly vulnerable population. Their comparatively small numbers compared to male offenders has meant that most programmes and services on the whole tend to be geared towards the majority male population. However women remandees represent female offenders, who have unique issues and needs different from their male counterparts, characterised by severe psychotic and neurocognitive disorders, predominantly non-violent offending, and absence of substance use. Clear differentiation between mental state at the time of offence and present fitness to plead is essential. This would be crucial in the future development of gender-specific programmes for incarcerated women orgender-responsive programming for female offenders. To do so, we would need to assess choices and attitudes that lead to the commission of the crime but also address these factors in the context of central issues specially for women, specifically, history of trauma and violence, substance abuse and economic marginality.

